# Global patterns of change and variation in sea surface temperature and chlorophyll a

**DOI:** 10.1038/s41598-018-33057-y

**Published:** 2018-10-02

**Authors:** Piers K. Dunstan, Scott D. Foster, Edward King, James Risbey, Terence J. O’Kane, Didier Monselesan, Alistair J. Hobday, Jason R. Hartog, Peter A. Thompson

**Affiliations:** 1 CSIRO Oceans and Atmosphere, Hobart, Australia; 2 CSIRO Data61, Hobart, Australia

## Abstract

Changes over the scale of decades in oceanic environments present a range of challenges for management and utilisation of ocean resources. Here we investigate sources of global temporal variation in Sea Surface Temperature (SST) and Ocean Colour (Chl-a) and their co-variation, over a 14 year period using statistical methodologies that partition sources of variation into inter-annual and annual components and explicitly account for daily auto-correlation. The variation in SST shows bands of increasing variability with increasing latitude, while the analysis of annual variability in Chl-a shows mostly mid-latitude high variability bands. Covariation patterns of SST and Chl-a suggests several different mechanisms impacting Chl-a change and variance. Our high spatial resolution analysis indicates these are likely to be operating at relatively small spatial scales. There are large regions showing warming and rising of Chl-a, contrasting with regions that show warming and decreasing Chl-a. The covariation pattern in annual variation in SST and Chl-a reveals broad latitudinal bands. On smaller scales there are significant regional anomalies where upwellings are known to occur. Over decadal time scales both trend and variation in SST, Chl-a and their covariance is highly spatially heterogeneous, indicating that monitoring and resource management must be regionally appropriate.

## Introduction

Understanding the spatial and temporal patterns of change in oceans is important for managing oceans by ensuring sustainable development and conservation^1,2^. Patterns of human use are modified by changes in weather and climate, which are occurring across scales of days to millennia. While changing climate is important over long time scales, most human activity and planning is confined to shorter time scales, typically less than a decade. Patterns of change and variability over years to decades will have a direct impact on the sustainability and livelihoods of all ocean-based activities (e.g.^3–5^). Understanding the observed spatial patterns, trends and variance of ocean states over these shorter time scales is important to complement other management, monitoring and planning efforts^5^.

Changes in ocean surface temperature are associated with changes in other variables, including biological variables such as Chl-a, primary productivity, species physiological responses and species distributions^3,6,7^. The patterns of temporal and spatial variability in Chl-a, and how it relates to temperature, directly link changes in climate with the dynamics of ocean ecosystems^1,3^. While the global average temperature is increasing^1^, there is variability around this average with different regions and locations experiencing different responses, both in terms of the trend and variance on different time scales^5,8,9^. The same will hold for changes in primary productivity and other ocean variables^3,10^. Understanding the pattern of variation at decadal time scales in important ocean variables, and how they covary, is a key component of our ability to adapt to variation in ocean state. While changes in long-term average conditions have received most attention, without assessment of decadal variability around these averages it is unlikely that we will be able to successfully respond to observed and predicted climate effects^5^. Short-term variation can overwhelm mean changes in many regions, leading to short term events such as marine heatwaves, changes in local productivity and ecosystem structure and changing the direction of the long-term trend^5^.

Sea surface temperature (SST) and chlorophyll a (Chl-a) are often linked^6,7,11,12^. Warming of the surface ocean increases temperature stratification in the upper ocean and can be associated with a reduction in surface mixing depth. Warmer temperatures tend to increase cellular Chl-a while increased irradiance or decreased nutrient availability tend to reduce cellular Chl-a^13–15^. Depending upon the magnitude of each factor phytoplankton cellular Chl-a will change (a response termed ‘photoacclimation’) (c.f.^6,7,11,12^). These changes in cellular Chl-a may not be related to a change in primary production. In some cases, a deepening of the mixed layer depth may increase the vertical transport of nutrients to the mixed layer potentially leading to increased Chl-a associated with an increase in phytoplankton division rates. There is growing evidence that in some locations increasing wind speeds overcome any potential increase in stratification due to warming and produce increased Chl-a^16,17^. These processes in the oceans are, however, variable on multiple spatial and temporal scales with significant impacts on ecosystem dynamics^18^. Understanding the patterns of annual variation (in SST and Chl-a, both individually and jointly) complements our understanding of change in SST and Chl-a across years.

In this work, we focus on analysis of daily global satellite observations from December 2002 to January 2015, for both SST and Chl-a. In particular, we seek to understand (1) seasonal patterns of variation, (2) the long term trend, and (3) the covariation between SST and Chl-a. We identify where inter-annual change is occurring and the extent of annual variation for both SST and Chl-a. The annual variation we estimate is a de-trended estimate of variation for each cell, which is in contrast to previous analyses (e.g.^19–21^). Those analyses quantify the variation within *binned regions* of the oceans and assume that there is no temporal change within each bin. In this work, we avoid this confounding by explicitly partitioning these sources of variation. We then contrast patterns of variation between SST and Chl-a and show that the observed patterns of covariation between SST and Chl-a demonstrate both positive and negative relationships suggesting SST alone may not be a good predictor of changes in Chl-a at global scales.

## Results

We analyse individual high resolution time series of SST and Chl-a (globally at 4 km^2^ grid cell resolution totaling 21024324 and 21024580 cells and models respectively) separately over the global ocean. We include only high quality data (i.e. where there is no cloud or solar reflectance) from the MODIS/aqua satellite sensor for the longest interval available. We do not rely on spatial interpolation, which avoids assumptions of spatial interpolation methods^22,23^. We only consider time series for cells that have more than 25 observations over the study period, for each of SST and Chl-a, and we assume that missing data are randomly distributed in time. Cells with less data than this tended to not have sufficient information to support estimation of all the model’s parameters (at any level of uncertainty). Note that cells with fewer observations will have greater uncertainty and may produce spatial regions where the signal is highly variable. The filtered data were quality controlled by searching for outliers, using a simplified yet robust version of the model. The model fitted, to each time-series, is a generalised additive mixed model (GAMM)^24^ with components for long-term trend, for seasonal patterns and for residual correlation between daily observations. For each cell and for SST and Chl-a, we produce summary metrics of the components of temporal variation in the time-series model, which are collated to produce maps^8,25,26^. See the Methods Section for more details and the Supplemental Material for an illustrated description of the process.

### Model Summaries

#### ALT

Average linear trend. This is the average rate of change throughout the study period after adjusting for potential non-linear relationships^8^. Positive values indicate a rise in SST (or Chl-a), negative values indicate a decline and values close to zero indicate no change on average.

#### Trend RMSE

A measure of the level of non-linearity of the inter-annual temporal effect. Values close to zero indicate that there is no non-linearity and larger values indicate increasingly non-linear responses.

#### Annual RMSE

A measure of the amount of seasonal variation. Small values (close to zero) indicate low seasonality and larger values indicate increasingly large seasonality.

#### GoF RMSE

A measure of goodness of fit (GoF) of the decomposition into an inter-annual trend and a seasonal trend. Large values of this statistic indicate that the decomposition is not supportable, and the seasonal pattern is not regularly repeating, and small values indicate the opposite.

### Variation in SST

**Figure 1:**
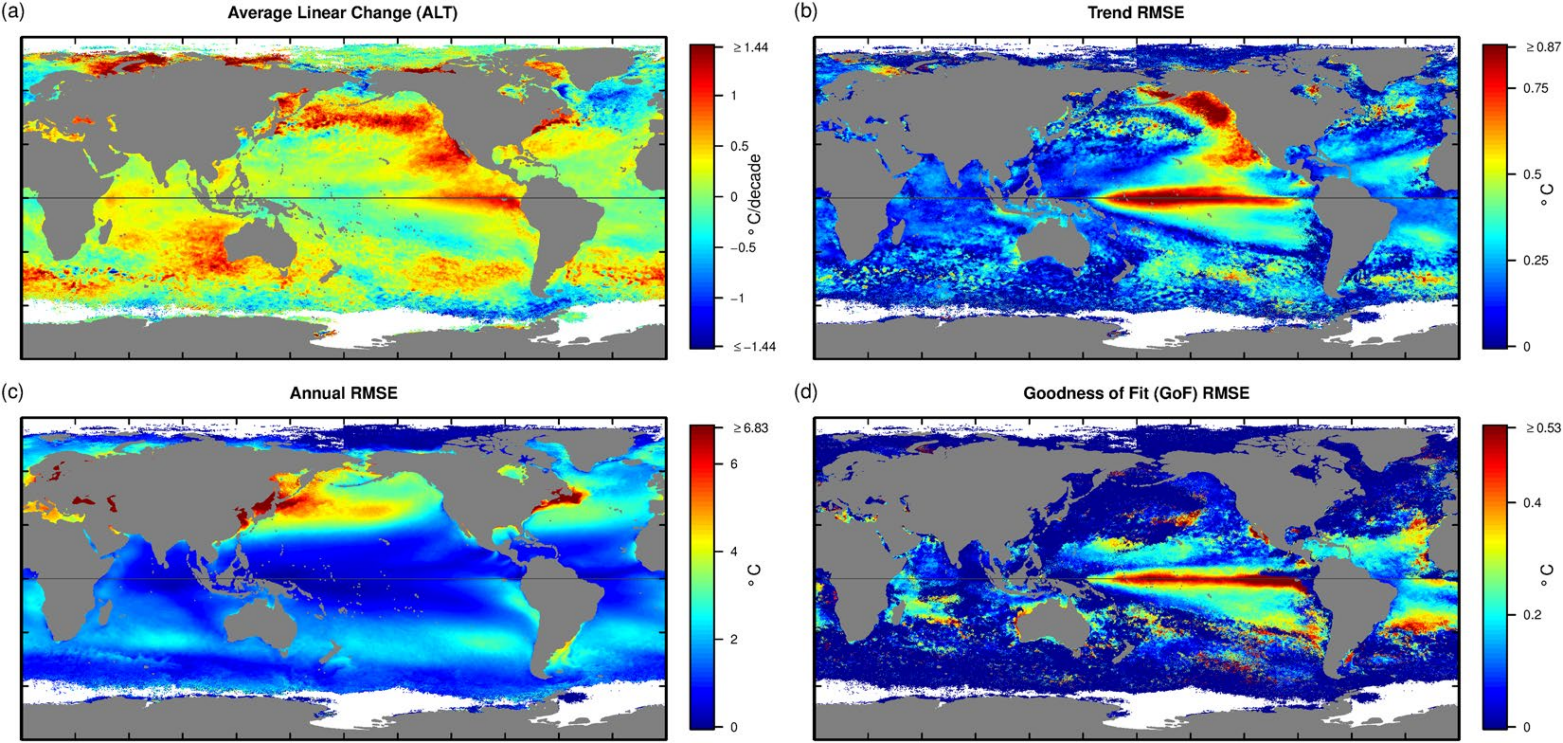
(**a**) ALT SST, the average linear trend over the study period (°C/decade). (**b**) Trend RMSE of SST, the measure of the magnitude of the non-linear component of the trend (higher values are more non-linear). (**c**) Annual RMSE for SST, the measure of the strength of the seasonal cycle (Annual RMSE, higher values indicate more seasonal variation). (**d**) GoF RMSE, the measure of how repeatable the seasonal cycle is from year to year (high values indicate more departures from a regular seasonal cycle).

The analysis of SST ALT (Fig. 1a) shows the warming of the Gulf Stream, and cooling of the North Atlantic, intense warming across the north Pacific consistent with the observed signal of the Interdecadal Pacific Oscillation (IPO), and warming patterns in the south-east Indian Ocean and Tasman Sea (off south-east Australia) over the time frame of the observations^27^. The SST Trend RMSE (Fig. 1b) highlights the IPO and El Nino Southern Oscillation (ENSO) signals within the observed time frame^9^ and the eddy fields in mid southern latitudes in the Indian, Pacific and Atlantic Oceans. However, it is likely that only a single phase of major climate drivers such as the IPO and IOD occur in the 14 year time series and the patterns may change when the IPO or IOD phases change. Annual RMSE (Fig. 1c) shows little annual variation in the central Pacific as all the variance in the central Pacific occurs in time scales longer than years. In contrast, there is intense annual variation in the north-west Pacific and north-west Atlantic. Of note are the eddy patterns derived from the Malvinas Current (east of Argentina; Location shown in Fig. S3, N), the areas of high variation associated with the Far East Pacific Fresh Pool^28,29^, and the Gulfs of Tehuantepec and Papagayo^30^ on the west coast of central America (Location shown in Fig. S3, J,K,L respectively). Annual variation is driven by rain and wind patterns^28,29^ in the Far East Pacific Fresh Pool and gap winds off the central American coastline^30^. The GoF RMSE (Fig. 1d) shows the spatial influence of non-seasonal signals, such as ENSO.

### Variation in Chl-a

**Figure 2:**
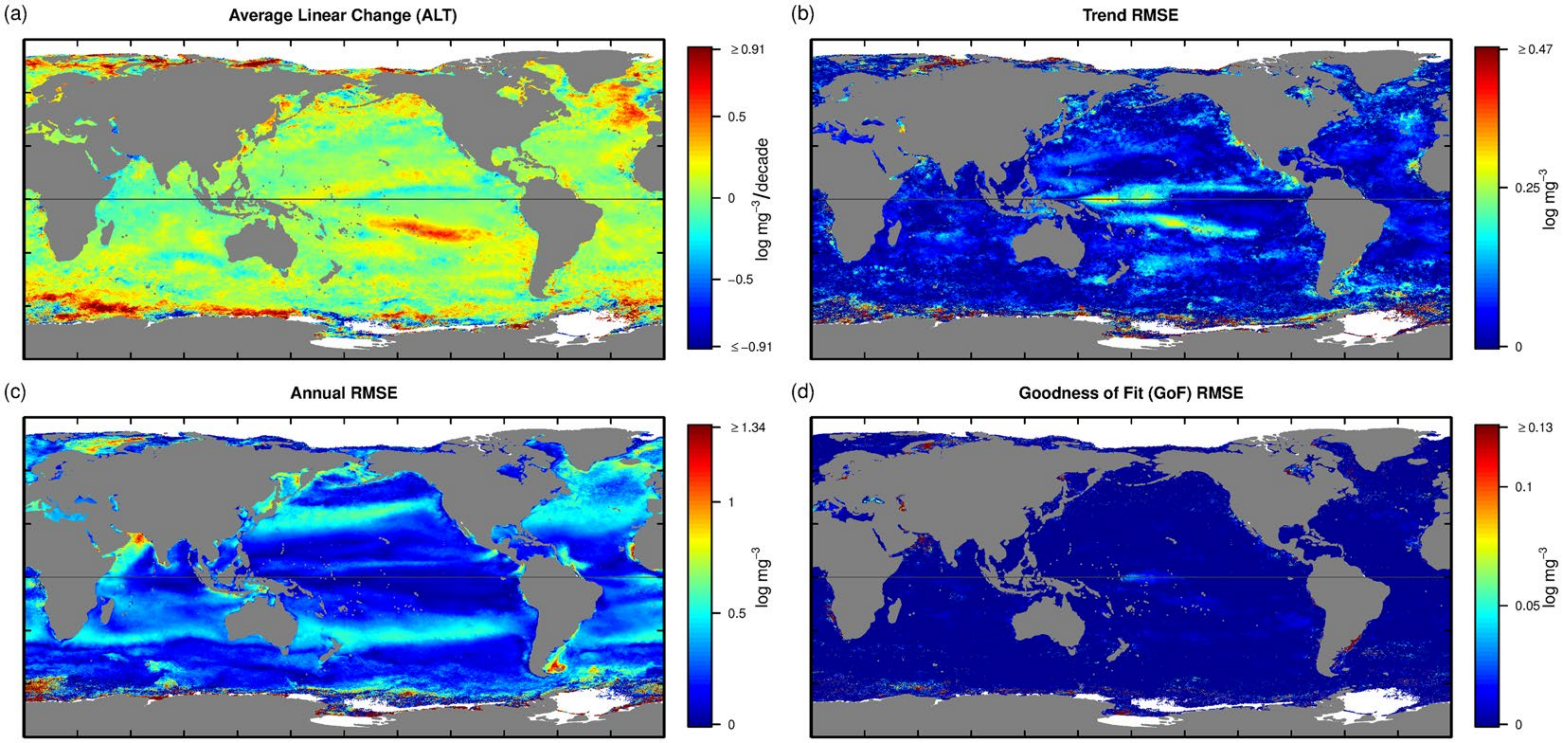
(**a**) ALT Chl-a, the average linear trend over the study period log(mg/m^3^/decade). (**b**) Trend RMSE, the measure of the magnitude of the non-linear component of the trend (higher values are more non-linear). (**c**) Annual RMSE, the measure of the strength of the seasonal cycle (higher values indicate more seasonal variation). (**d**) GoF RMSE, the measure of how repeatable the seasonal cycle is from year to year (high values indicate more departures from a regular seasonal cycle).

**Figure 3:**
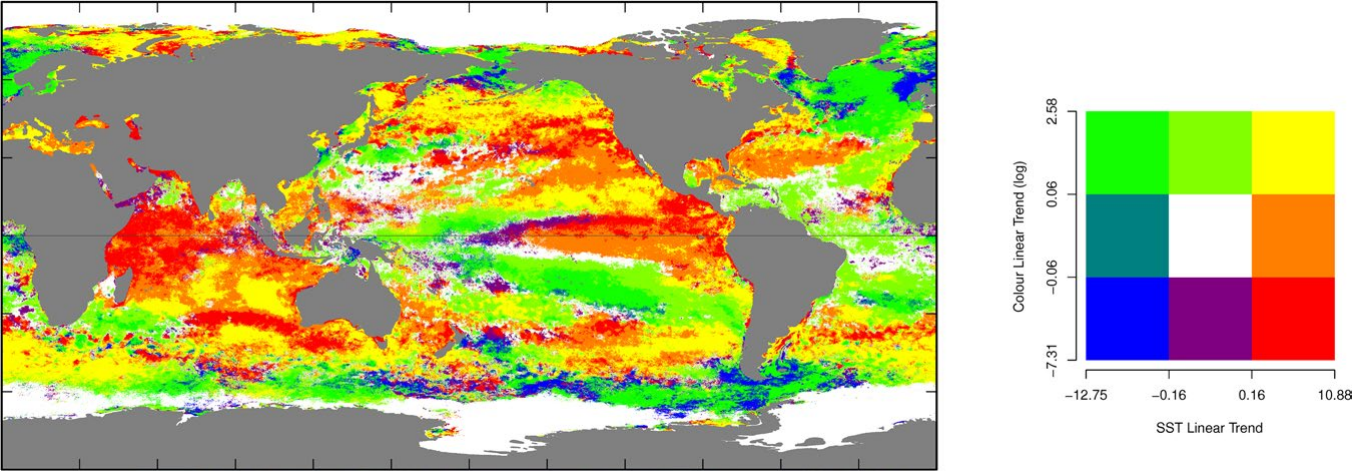
Magnitude and direction of the linear trend (ALT) in SST and Chl-a (units are °C/decade for SST and log(mg/m^3^/decade) for Chl-a).

Patterns for Chl-a (Fig. 2) show substantially different patterns from SST. The linear trend (ALT; Fig. 2(a)) shows localised patterns of increases and decreases in Chl-a, within the time frame of observations. There are substantial decreases in Chl-a north west of the United Kingdom, surrounded by an area of increasing Chl-a. There is a strong increase in Chl-a along the southern boundary of the South Pacific Convergence Zone (SPCZ), a band with precipitation of ~2 metres per year stretching from Papua New Guinea east and south towards ~20°S and ~150°W suggesting a southward movement of Chl-a possibly associated with photo-physiology associated with shoaling of the mixed layer or more nutrients in the euphotic zone in this generally nutrient-limited area. Other notable features include the standing eddy fields south of Africa (Aghulus retroflection, Location shown in Fig. 3B) and the Malvinas Current, both of which are also visible in SST ALT (Fig. 1(a)). The Trend RMSE (Fig. 2b) has very little signal, with exception of the Pacific Warm pool which intensifies with ENSO La Nina events.

Areas of strong annual RMSE for Chl-a (Fig. 2c) appear as bands in the transition zones between the subtropical gyres and the subpolar gyres (e.g. the Southern Hemisphere along the northern boundary of the subtropical front). Irregular seasonal variation in the location of these fronts appears to have a significant impact on Chl-a over a substantial portion of the oceans. When a positive Southern Annular Mode (SAM) aligns with La Nina events eddy kinetic energy increases significantly along the subtropical front^31^ potentially stimulating blooms of coccolithophores^32^ during austral summer (December-January). At a smaller scale the variable seasonality in the Gulfs of Tehuantepec and Papagayo, the Far Eastern Pacific Fresh Pool and Malvinas Current are apparent and consistent with anomalous seasonal warming (Fig. 2(c)). There are significant responses in Chl-a found in association with the Canary Current, the Arabian and Red Sea, Barents Sea, the southern Java Upwelling^33^ and the Sea of Japan and Okhotsk (Location shown in Fig. S3, O,B,C,A,F,G,I respectively). The Arabian Sea and the coasts of Oman and Yemen showed a declining trend in Chl-a (Fig. 2a), despite very strong annual variation (Fig. 2(c)) in an area where seasonal upwelling is associated with strong winds during the southern summer monsoon season. These regions of upwelling are known to be susceptible to the frequency, intensity and timing of the Indian Ocean Dipole (IOD) and ENSO events^34^ which will contribute to the large variability observed in the Annual RMSE over the analysis period (Fig. 2c). Relative to SST a greater proportion of the temporal variability in Chl-a is described by the long term trend and seasonal variability possibly reflecting the fact that there are other important drivers of Chl-a such as insolation. Unlike SST, most of the variability in Chl-a can be captured in long term variability and annual variability (Fig. 2d) suggesting that Chl-a is more predictable between years than SST^35^.

### Relationships between SST and Chl-a

**Figure 4:**
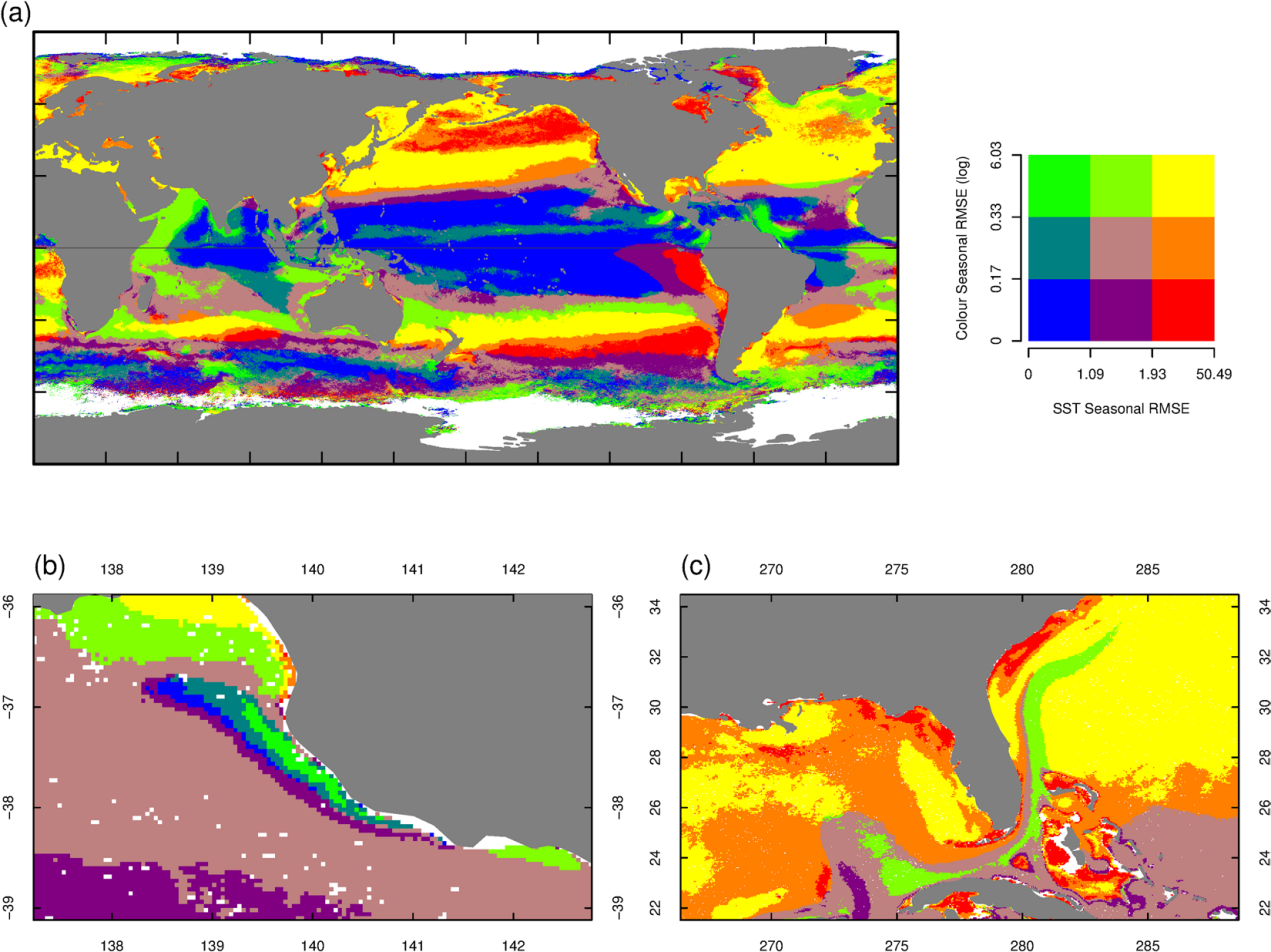
(**a**) Annual variation (Annual RMSE) of SST and Chl-a globally (units are °C/decade for SST and log(mg/m^3^/decade) for Chl-a). (**b**) The pattern of annual variation in the Bonney Upwelling, Southern Australia. (**c**) The pattern of annual variation in the the Florida Current, South East USA.

The correlations between SST and Chl-a trend and variability have rarely been explored on a global scale. Previous analyses (e.g^6,7,19–21^.) have used linear regressions and standard deviations on binned areas which will not explicitly partition the inter-annual, seasonal, and daily components of temporal variance at a cell level. At worst, this approach may generate biased estimates due to confounding of the components of variance. We compare the global spatial distribution of the ALT and Annual RMSE summary statistics for each 4 km^2^ cell for both SST and Chl-a (Figs 3 and 4). The long term trend over 12y can, potentially, reflect a mix of localized processes and the slow advection of water masses. This analysis does not consider the instantaneous covariance between SST and Chl-a. Rather, it considers the relationships between trends and patterns of annual variance. The implication of this is that the model does not explicitly allow for advective processes that could introduce lagged dependencies between SST and Chl-a. This could only be overcome in future applications by proposing a full bivariate statistical spatio-temporal model with complex covariance structure defined by coupling through advection and other processes.

There are areas of intense warming combined with decreasing or stable Chl-a in the central and north Pacific, the Sargasso Sea (Fig. S3, M) and Gulf Stream and across most of the Indian Ocean (Fig. 3). The most likely mechanism for this combination of changes (increasing SST and decreasing Chl-a) is more intense stratification and a deepening of the thermocline resulting in reduced nutrient input into the euphotic zone^36^. This interpretation is consistent with the observations and modelling of the northern Indian Ocean indicating an increased depth of the 20 °C isotherm^37^ for (increasing SST and decreasing Chl-a) portions of the Indian Ocean. South of the area of warming associated with ENSO in the Pacific Ocean is an extensive area extending from South America toward Papua New Guinea where SST is decreasing and Chl-a is increasing, suggesting a southward movement of water masses^38^. The North Atlantic is generally cooling and increasing in Chl-a, with the exception of the west coast of Ireland and the UK, where the ocean is both cooling and decreasing in Chl-a. The downturn in temperature is considered to be a fluctuation in the Atlantic Meridional Overturning Circulation (AMOC) driven by broad scale natural climate cycles^39^. There are also significant areas where warming is apparent and Chl-a is increasing, which are often adjacent to the areas influenced by the IPO, ENSO and IOD. Also apparent are the eddy fields in the Aghulus Current, which show alternating patterns of warming and decreasing Chl-a and cooling and increasing Chl-a as seen in previously observed coarser global patterns^7^. Such patterns of covariation are consistent with greater nutrient delivery to the surface in cooler waters or a gradual change in location. While there are some patterns that are similar to previous studies (e.g. Sargasso Sea, parts of the central Indian Ocean, central Atlantic^6,7,19–21^), there are significant differences in every other region of the ocean (e.g., central Pacific, north Pacific, western Indian Ocean, Bay of Bengal (Fig. S3, E), north Atlantic, Southern Ocean). Similar negative correlations between increasing SST and decreasing Chl-a trends in the north west Indian Ocean have been previously observed^36^. While there are some similarities in the trends, our analysis shows areas where Chl-a is trending up and where SST is decreasing and Chl-a is trending down and SST is increasing. These differences are due to differences in both duration of time series data (our period is twice as long), potentially different mode of ocean states, use of a different statistical model (ours allows an estimation of variance) and a different satellite platform.

## Discussion

The interaction between annual variation in SST and Chl-a provides insights into how and where linkages occur on annual time scales. Our analysis shows strong latitudinal bands associated with variation in seasonal warming (Fig. 4a). The equatorial Pacific, Indian and Atlantic Oceans are all characterised by very low annual RMSE for both SST and Chl-a. The mid latitudes of each ocean basin have higher variance in SST and/or Chl-a. However, the Southern Ocean shows more complex patterns, with areas of very low variance adjacent to areas of much higher variance in SST and/or Chl-a, reflecting the close proximity to each other of the various polar fronts. While the ocean basins show very large scale patterns, areas adjacent to the coasts in all the basins show a more complex pattern. This is most clearly shown by the areas of high variance in the Far East Pacific Fresh Pool, and the Gulfs of Tehuantepec and Papagayo where the link between Ekman pumping on the margin of the Eastern Tropical Pacific and seasonal variation in Chl-a and SST is notable and the western boundary of the Indian Ocean. These areas are surrounded by larger regions of low variance in low latitudes. Examining the outputs in fine detail can show patterns such as the Bonney Upwelling (Figs 4b and S3, H) on the southern coast of Australia, and the Florida Current (Fig. 4c), the expression of the Gulf Stream visible around Florida.

The patterns revealed here provide both a snapshot and evidence for change and variation over the 14 year period. Numerous studies have shown that changes in ocean temperature are already affecting the distribution of marine life (e.g.^40–42^) while changes in chlorophyll standing stocks and ocean productivity also have dramatic impacts^43^. Our analysis shows that both SST and Chl-a are changing globally and the covariance depends on location. It is likely that additional change, coupled to variation around that trend, will occur, and continue to affect fisheries^44^, the health of ecosystems (e.g. coral bleaching frequency and severity^45^), the dynamics of ecosystems^46^ and extreme weather events (e.g. marine heatwaves^47,48^). These patterns can inform understanding of the observed and projected changes in marine ecosystems around the world, and improve understanding of the likely responses to climate change at the spatial and temporal scales governing human interactions with marine resources. Identification of these patterns can guide management responses such as harvesting levels for fish, monitoring and data collection, inform landscape level planning activities such as marine spatial planning and bioregionalisation and additional process studies that can compare biological responses to change in different regions over different time scales.

## Methods

The SST data were derived using the longwave SST algorithm NASA Goddard Space Flight Center (2014) from brightness temperature data recorded by the MODIS/Aqua satellite sensor, and obtained from the NASA Ocean Biology Processing Group (http://oceandata.sci.gsfc.nasa.gov). The advantage of using MODIS/Aqua as the sensor for the SST is that the observations are exactly simultaneous with the Chlorophyll-a measurements and have the same field of view and cloud masking, enabling a more straightforward comparison than if the two data sets had been recorded at different times by different sensors. Level 3 daily netcdf-format files with global coverage mapped at 4 km spatial resolution (10.5067/AQUA/MODIS_OC.2014.) were downloaded for the period 2002-07-04 through 2016-01-15. The daily data were concatenated to create a third dimension (time), and then internally re-ordered to provide efficient access along the time dimension to facilitate the time series analysis.

The Chlorophyll a observations were also derived from radiances measured by the MODIS/Aqua sensor and the data were obtained from the same source and in the same format as the SST data. The product used was derived using the OCI algorithm^49^ and subject to the same reorganisation as the SST data.

### Which Cells to Analyse?

For all satellite-derived variables, we analyse only those cells with more than 25 observations. We note that cells with few observations may produce highly variable models, parameter estimates and ultimately inferences. The number of observations per cell required here is less than that required in Foster *et al*. ^8^. This is because Foster *et al*.^8^ considered only a mid-latitude region which coincidentally avoided areas of high solar reflectance (low latitudes) and oblique observing angles (high latitudes). If any information is to be provided for very low and very high latitudes, then the less stringent criterion must be used (at the expense of increased uncertainty even though it is quantified here). Gross inadequacies in this criterion may be evident in the model output as highly spatially variable regions.

### Model

The temporal data from each cell are analysed as a time-series. The resulting models are then summarised and collated over all cells to produce maps of meaningful data-driven quantities.

#### Identifying Outliers

All data are susceptible to contamination by artefacts and remotely sensed data is no exception. Here, like in Foster *et al*.^8^ and elsewhere, we take a pragmatic approach and remove potential outliers prior to performing the formal analysis.

Due to global scope of the analyses, the method used to detect outliers described in Foster et al.^8^ was slightly modified. We assess each datum’s chance of being an outlier by inspecting it in relation to a trimmed mean (for example, see^50^). The trimmed mean is a robust estimator of the mean of the central (in distribution) 90% of the nearest observations in time. We use the nearest 25 (12 on each side in addition to the current observation) for SST and Chl-a. This method of generating a robust expectation is not dependent on a common seasonal cycle, unlike that used in Foster *et al*.^8^, and will naturally adjust itself when the seasonal cycle varies depending on other pressures (e.g. an ENSO cycle). It will also naturally adjust the smoothing window depending on data density – those cells with few data will be more heavily smoothed than those cells with more data, but only for detection of outliers.

The task remaining is to define a rule for determining how removed from the running trimmed mean an observation actually is. For data that is usefully considered Gaussian (SST) we consider that any datum, *y*_*t*_, that lies outside *µ*_*t*_ ± 3.89 × *σ*_*m*_ is an outlier. This interval is the central 99.99% interval of a Gaussian distribution defined by the running trimmed mean at time *t*, *µ*_*t*_, and (robust) standard deviation *σ*_*m*_. The robust standard deviation is calculated using the method of mean absolute deviations (see^50^). For data that can be usefully considered to be gamma distributed (Chl-a) we take the central 99.99% interval again, with the gamma distributions scale parameter estimated from the data, given the running trimmed mean (see^50^). Note that this is not a robust estimate of scale and so the outliers will cause slightly inflated dispersion estimates. The effect of not having a robust measure is that the outlier detection method may be slightly more tolerant of outliers.

#### Time-series Models

The time-series model attempts to decompose the data into a small number of components of variation. It is applied to the data from each cell and to each measurement variable. The model is useful, as the components can be made to mirror common temporal sources: notably day-to-day variation, seasonal variation and longer-term variation. The model is based on that presented in Wood^24^ (Section 6.7) with only minor alterations, and is similar to models used previously for other environmental monitoring applications (for example see^51–53^). The components of variation considered are:

Inter-annual: This includes all variation, which is smooth through time, which occurs on a time scale greater than one year. This is modelled through a smoothing spline term (a smoothing spline) *f*(*t*) where *t* is the number of days since the time-series began. The smoothing spline is partially defined through its knot points (defined prior to analysis) and its smoothing parameter (defining the ‘wiggliness’ in the smooth). Unlike Foster *et al*.^8^, we fit two models that have different wiggliness in their inter-annual spline. The first, *f*_1_(*t*), with a knot point for each year, is not flexible enough to change substantially within a year. Thus, this spline can only detect changes that are smooth in multiple years. This spline mirrors that presented in Foster *et al*.^8^. The second, *f*_2_(*t*), has many more knot points and is capable of detecting *within year smooth departures that are not common to all years*. The maximal number of knot points considered is min(101,*n*_*i*_) where *n*_*i*_ is the number of available observations for the cell under consideration. The function *f*_2_(*t*) will be flexible enough to model inter-annual *and* within-year patterns that are not attributable to seasonal variation (see below). Thus, inspecting the difference (across time) of *f*_1_(*t*) and *f*_2_(*t*) provides a check on how ‘seasonal’ the time series actually is (does the same seasonal pattern repeat year after year).

Seasonal: This is a periodic function that captures the annually repeating pattern in the time series. Its value on the 31^st^ of December matches that on the 1^st^ of January (along with its first and second derivatives). We label this function *g*(*d*), where *d* is the day of the year (ranging from 1 to 366), and model it using a cyclic spline^24^. We specify 9 knot points, which is large enough to provide considerable flexibility but also small enough to reduce unnecessary computational burden.

Day-to-day (residual): All departures of the observed data from the functions defined by the inter-annual signal (*f*_1_(*t*) or *f*_2_(*t*)) and the seasonal signal (*g*(*d*)). It includes: randomness in the day-to-day measurements (including measurement error), known sources of variation that have not been included in the model (such as diurnal patterns), and non-smooth sources of variation. We assume that these terms follow a Gaussian distribution for SST and a gamma distribution for Chl-a. These choices were made after inspecting the time-series for some example locations. In addition, we assume that the departures from expectation are correlated and are described using a first order autoregressive process. To ease computational burden, we make the assumption that correlation only exists within a year and that correlation between days in different years is zero^8,24^.

Formally, these three components of variation can be incorporated into the model

$$h({\rm{E}}(y(t,d)))={f}_{j}(t)+g(d)$$

where *f*_*j*_(*t*) is either *f*_1_(*t*) or *f*_2_(*t*) and is the inter-annual trend, *g*(*d*) is the seasonal cycle, E(·) is the expectation operator and *h*(·) is a link function. For Gaussian data we specify *h*(·) to be the identity link and for gamma data, we specify *h*(·) to be the log link. The log link function ensures that the expectation for the data is kept positive. The observed data are distributed around this expectation.

For Gaussian data, we estimate the model parameters using restricted maximum likelihood^24,54^. For Gamma data, the likelihood is not well defined (as correlated gamma variables cannot be defined as above) and penalised quasi-likelihood (PQL) is used^24,55^. The PQL method uses a working likelihood in estimation, which is Gaussian and so can be specified with the correlation parameter.

#### Further Detail of Model Summaries

ALT: Average linear trend. This is the average rate of change throughout the study period after adjusting for potential non-linear relationships^8^, by exploiting the representation of a spline as a straight line plus smooth deviations from it^24,56^. The ALT summary is the slope of the straight line component.

Trend RMSE: A measure of the level of non-linearity of the inter-annual smoothing spline, the measure of inter-annual variation. Calculated as the root mean square error (RMSE) between the inter-annual smooth and the ALT, that is Trend ${\rm{RMSE}}=\sqrt{\frac{1}{N}{\sum }_{t=1}^{N}{[{f}_{1}(t)-ALT(t)]}^{2}}$ where *N* is the number of days in the study and ALT(*t*) is the average linear trend calculated at time *t*.Annual RMSE: A measure of the amount of seasonal variation, as encapsulated in the estimated function *g*(*d*). Calculated as the RMSE between the cyclic seasonal function and zero, that is annual ${\rm{RMSE}}=\sqrt{\frac{1}{366}{\sum }_{d=1}^{366}{[g(d)]}^{2}}$. Annual RMSE is a de-trended estimate that removes the influence of the inter-annual trend (*f*_*j*_*(t)*).

GoF RMSE: A measure of goodnes of fit (GoF) of the decomposition into smooth inter-annual trend and seasonal trend is. Put another way: how much evidence is there that there the seasonal cycle is dependent on less predictable pressures? It is calculated as the RMSE between the inter-annual smooth *f*_1_(*t*) and its more flexible (allowing for intra-annual variation) counterpart *f*_2_(*t*). That is, GoF ${\rm{RMSE}}=\sqrt{\frac{1}{N}{\sum }_{t=1}^{N}{[{f}_{1}(t)-{f}_{2}(t)]}^{2}}$, where *f*_1_(*t*) and *f*_2_(*t*) are defined as the less flexible smooth and the more flexible smooth respectively.

#### Assumptions and Model Limitations

Missing data are assumed to be missing at random throughout the study period. If not then there could be bias in results. For example, if winter measurements are more likely to be missing then the average temperature will be biased upwards and the amount of seasonal variation likely to be biased downwards. Similar arguments can be made for: 1) changing raw data (satellite) processing algorithms so that later measurements are different to earlier ones, or 2) changing atmospheric conditions that made the measurement (but not the true state) appear different.

The ALT and other summaries based on *f*_1_(*t*) may be unreliable in those locations where the seasonal pattern does not regularly repeat. In these locations, the GoF RMSE will be inflated and can be used as a reasonable diagnostic.

## Data Sets

The datasets generated during the current study are available in the Marlin repository at http://www.marlin.csiro.au/geonetwork/srv/eng/search?uuid=c685a21e-8770-4b3b-ac3c-2c4f815f7176.

## Electronic supplementary material

Supplementary Information
