# Supplementary material for: Global patterns of change and variation in sea surface temperature and chlorophyll a
Source: Sci Rep. 2018 Oct 2;8:14624. doi: 10.1038/s41598-018-33057-y (PMC6168485; doi:10.1038/s41598-018-33057-y)

Global patterns of change and variation in sea surface temperature and chlorophyll a

Authors: Piers K. Dunstan^1^, Scott D. Foster^2^, Edward King^1^, James Risbey^1^, Terence J. O’Kane^1^,

Didier Monselesan^1^, Alistair J. Hobday^1^, Jason R. Hartog^1^, and Peter A. Thompson^1^

^1^CSIRO Oceans and Atmosphere

^2^CSIRO Data61

Supplemental Material

The analysis steps, and model summaries, can largely be illustrated graphically. This is done for two example locations in Fig S1 and S2. In both figures, panel (a) gives the outlier detection method where a running trimmed mean is applied to the data to get a measure of location. Those points that are far removed from the running trimmed mean line are considered outliers. The Central Pacific location (Fig S1) has a non-linear trend in the time-series (declining and the increasing) but with many exceptions to this general rule (Fig S1c), although its linear trend is not substantial. The modelled pattern, which assumes an annually repeating seasonal cycle, does not capture the variation in parts of the time series (Fig S1d), most notably around 2010). This gives rise to an inflated GoF statistic indicating that other drivers, apart from seasonal and smooth inter-annual, are affecting the signal. The Maria Island example location (Fig S2) contrasts the Central Pacific location in many ways. At Maria Island: there is a strong repeating seasonal cycle (Fig S2b and S2c); the long-term trend is largely linear (except for the beginning and end of the data, Fig S2c); There is a positive, but not very large, average linear trend (Fig S2c), and; there appears to be no long-term drivers apart from the modelled long-term trend (Fig S2d).

Fig S1. Visual guide to analysis steps for a location in the central pacific. Panel (a) demonstrates the detection and removal of outliers from the time series using a robust estimate of trend. The modelled seasonal cycle (b) and the long-term trend (c) from the fitted GAMM. Note that (b) has a reference line included for when there is no seasonal cycle, and (c) has had the average linear trend (ALT; calculated from the non-linear trend) added. The more flexible fit is compared in panel (d) and is shown against the more constrained time-series model. The summary statistics are: ALT, which is the slope of the black line in panel (c); Trend RMSE, which is given by the RMSE between the red line and black lines in panel (c); Annual RMSE, which is the RMSE between the blue and black lines in panel (b), and; GoF RMSE, which is the RMSE between the blue and red lines in panel (d).

Fig S2. As per Fig S1 but for a location near Maria Island off the East Coast of Tasmania, Australia.

Figure S3.

Illustrative map of regions identified within the manuscript


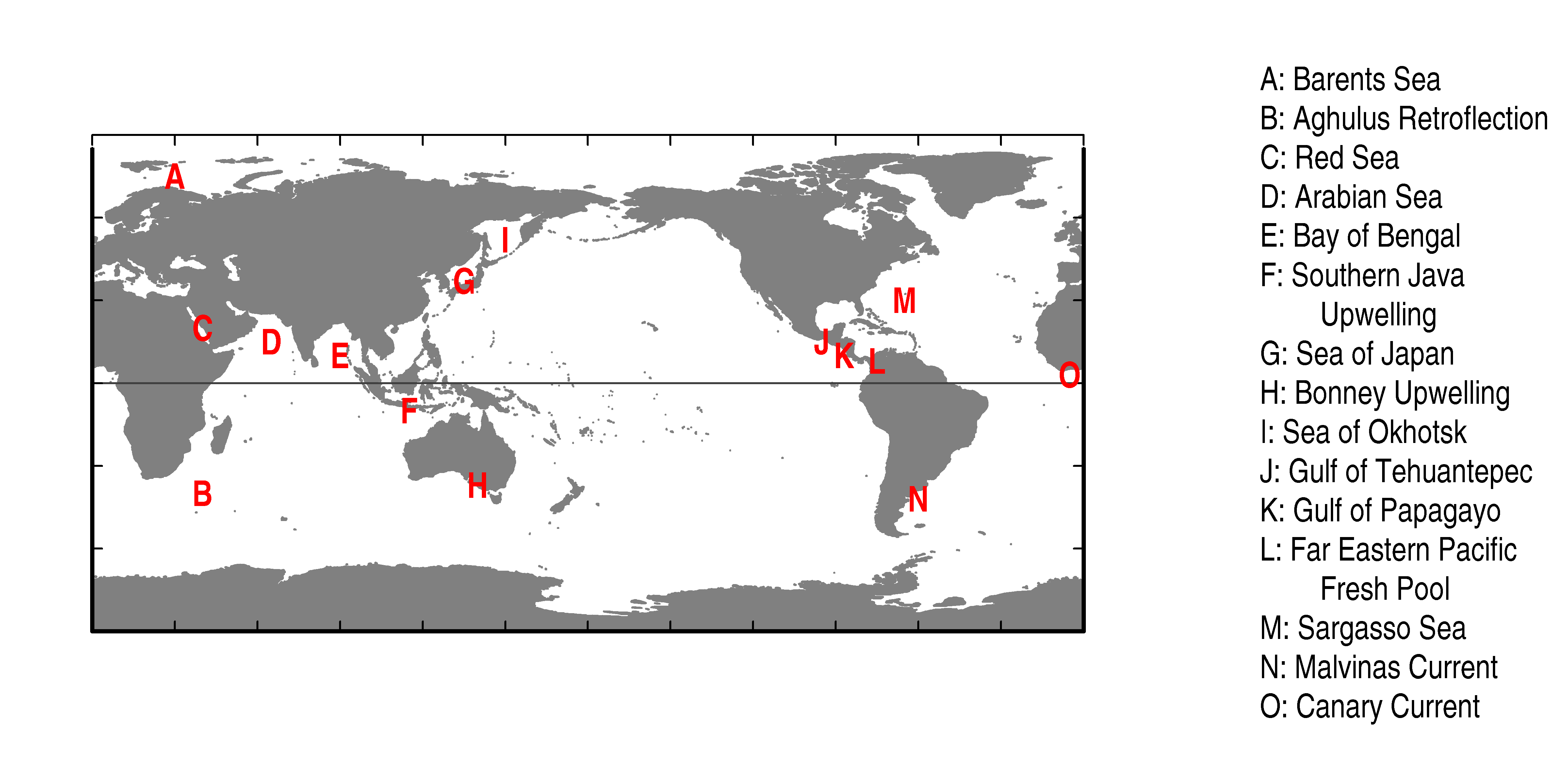

Supplement: Supplementary file 1 — Supplementary Information [file 41598_2018_33057_MOESM1_ESM.docx]
